# INS/GNSS Tightly-Coupled Integration Using Quaternion-Based AUPF for USV

**DOI:** 10.3390/s16081215

**Published:** 2016-08-02

**Authors:** Guoqing Xia, Guoqing Wang

**Affiliations:** College of Automation, Harbin Engineering University; Harbin 150001, China; xiaguoqing@hrbeu.edu.cn

**Keywords:** unscented particle filter, INS/GPS integration, USV, tightly-coupled integration, quaternion

## Abstract

This paper addresses the problem of integration of Inertial Navigation System (INS) and Global Navigation Satellite System (GNSS) for the purpose of developing a low-cost, robust and highly accurate navigation system for unmanned surface vehicles (USVs). A tightly-coupled integration approach is one of the most promising architectures to fuse the GNSS data with INS measurements. However, the resulting system and measurement models turn out to be nonlinear, and the sensor stochastic measurement errors are non-Gaussian and distributed in a practical system. Particle filter (PF), one of the most theoretical attractive non-linear/non-Gaussian estimation methods, is becoming more and more attractive in navigation applications. However, the large computation burden limits its practical usage. For the purpose of reducing the computational burden without degrading the system estimation accuracy, a quaternion-based adaptive unscented particle filter (AUPF), which combines the adaptive unscented Kalman filter (AUKF) with PF, has been proposed in this paper. The unscented Kalman filter (UKF) is used in the algorithm to improve the proposal distribution and generate a posterior estimates, which specify the PF importance density function for generating particles more intelligently. In addition, the computational complexity of the filter is reduced with the avoidance of the re-sampling step. Furthermore, a residual-based covariance matching technique is used to adapt the measurement error covariance. A trajectory simulator based on a dynamic model of USV is used to test the proposed algorithm. Results show that quaternion-based AUPF can significantly improve the overall navigation accuracy and reliability.

## 1. Introduction

The development of unmanned surface vehicles (USVs) for scientific, military and commercial purpose in applications such as oil and gas exploration, oceanographic data collection, hydrographic, oceanographic and environmental survey, mine counter measure, surveillance and reconnaissance, anti-submarine warfare and fast inshore attack craft for combat training require the corresponding development of navigation systems [1]. The need of the robustness, accuracy and reliability is a guarantee of long duration, unmanned operations for USVs. The sensor units on board, which serve as environment perceptions, are of critical importance for automation operation. A reliable and accurate navigation system is very important for a USV.

The integration of Global Navigation Satellite System (GNSS) and Inertial Navigation System (INS) is widely used for positioning and attitude determination for vehicles. The development of micro-electromechanical system (MEMS) technology has brought low-cost INS/GNSS integration approaches into practice [2,3]. A growing number of research groups are developing integrated navigation systems utilizing INS and GNSS due to the complementary nature of INS and GNSS principles. The INS/GNSS integration can be classified into loosely-coupled, tightly-coupled and deeply-coupled [4]. For the loosely-coupled manner, independent redundant solutions are available from the GNSS receiver and INS process. However, the disadvantage is that typically four satellites have to be in view to obtain position and velocity solutions from the GNSS receiver. In addition, cascaded filtering problems may occur when one local Kalman filter (KF) is used in GPS data processing and the other is used in integration. These problems can be easily solved in tightly-coupled integration. In tightly-coupled integration, a centralized KF is employed. The pseudorange and delta pseudorange (or Doppler) measurements are used as observations to update the navigation filter. Furthermore, the system does not need a full GNSS solution to assist the INS. This means that the error correlation of inertial measurement unit (IMU) measurements can maintain the update of the estimated solution by the INS even if the number of the tracked satellites is less than four. However, the resulting system observation model turns out to be nonlinear, which should be carefully treated in the design of the integration KF.

The EKF (extended Kalman filter) is known as state-of-the-art for fusion INS and GNSS data in tightly-coupled integration. The linearization should be implemented in both the nonlinear system model and the observation model first in order to apply EKF. This approximation will result in large errors when EKF calculates a posterior mean and covariance, which may lead to suboptimal performance or even divergence of the filter. UKF (unscented Kalman filter) is a recursive MMSE (Minimum Mean Square Error) estimator based on optimal Gaussian approximation Kalman filter framework. Unlike the EKF, the UKF uses the true nonlinear model and approximates the distribution of the state random variables [5]. The state distribution in the UKF is specified using a minimal set of deterministically chosen sample points to capture the posterior mean and covariance accurately to 2nd order for any nonlinearity. It is based on the assumption that it is much easier to approximate a Gaussian distribution than to simulate an arbitrary nonlinear function [6]. Recently, with the development of the computer technology, particle filter (PF) turns out to be more attractive for nonlinear and non-Gaussian applications, and has been successfully used in [7] to recursively update the posterior distribution by sequential importance sampling and resampling. However, the large computational burden impeded the practical use of PF. In addition, the sample impoverishments accompanying the degeneracy of the system performance are primary disadvantages of the basic PF [8]. To overcome these problems, in [3], the strategy of combining the UKF with PF was proposed.

To achieve better performance from the KF framework, the stochastic information provided to the filter must be as accurate as possible. It is therefore necessary to adapt the stochastic model to accommodate for changes in vehicle dynamics and environment conditions. Insufficient or incorrect knowledge about statistics may lead to degradation of the system performance or even divergence of the filter. In [9], a filter innovation sequence based on adaptive Kalman filtering (AKF) was introduced, which showed a major improvement in adjusting process noise error covariance and sensor measurement error covariance adaptively. In this paper, an adaptive unscented particles filter (AUPF) algorithm based on quaternion is proposed using a residual-based covariance matching technique.

In the remainder of this paper, the content is organized as follows. In Section 2, a trajectory simulator with corresponding sensors measurements allocated in a USV is developed. In Section 3, quaternion-based propagation and an observation model are introduced. Furthermore, a quaternion-based GPS/INS integration algorithm using AUPF is proposed. Simulation results are analyzed and compared to illustrate the performance of the proposed AUPF algorithm.

## 2. Sensor Measurement Simulation

**Figure 1:**
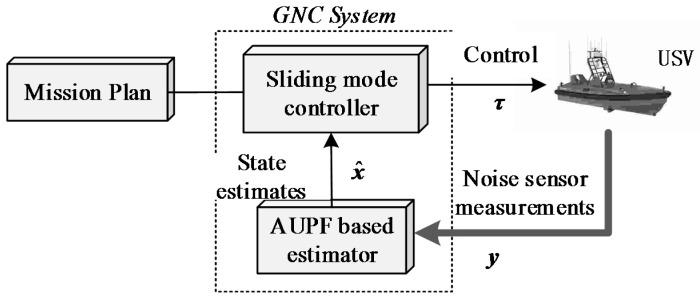
Schematic diagram of the simulation system.

In this part, a USV trajectory simulator is designed. We briefly summarize the different component parts of the USV platform. These include the mathematical dynamic state-space models used for the simulator, the sensors subsystem and the controller design. The core components of such a simulator are schematically depicted in Figure 1, which include a control system, a guidance system and a navigation system (GNC). The GNS system takes the noisy GPS and MEMS-IMU measurements as inputs, and then fuses them from the dynamics model of USV to estimate the optimal vehicle navigation state solutions. There state estimations together with desired trajectories from guidance systems are then adopted by the controller, which generates an optimal control law to drive the thruster of the USV.

### 2.1. Trajectory Simulator of the USV

As in [10], the marine craft equations of motion in six degrees of freedom can be written in vectorial setting form:

(1) $$\dot{\eta }=J_{\Theta }\left(\eta \right)\nu$$

(2) $$M\dot{\nu }+C\left(\nu \right)\nu +D\left(\nu \right)\nu +g\left(\eta \right)=\tau_{wave}+\tau_{wind}+\tau_{currents}+\tau$$

where $\eta \in {\text{ℝ}}^{6}$ denotes the position and the orientation vector. $\nu \in {\text{ℝ}}^{6}$ denotes the linear and angular velocity vectors that are decomposed in the body-fixed reference frame. $\tau \in {\text{ℝ}}^{6}$ describes the forces and moments acting on the craft in the body-fixed fame. The generalized position, velocity and force vectors have the form that represented as Equation (3):

(3) $$\begin{array}{ccc} \eta =\left[\begin{array}{c} p_{b/n}^{n}\\ \Theta_{nb} \end{array}\right], & v=\left[\begin{array}{c} \upsilon_{b/n}^{b}\\ \omega_{b/n}^{b} \end{array}\right], & \tau =\left[\begin{array}{c} f_{b}\\ m_{b} \end{array}\right] \end{array}$$

The same notation as in [10] is used, $p_{b/n}^{n}\in {\text{ℝ}}^{3}$ is the position expressed in the north east down (NED) frame, {N}. $\Theta_{nb}\in S^{3}$ represents the Euler angles. $\upsilon_{b/n}^{b}$ and $\omega_{b/n}^{b}$ represent the linear and angular velocity of body frame expressed in{B} frame with respect to {N} frame. $f_{b}\in {\text{ℝ}}^{3}$ and $m_{b}\in {\text{ℝ}}^{3}$ are the forces and moments acting on the vehicle, respectively. ${\text{ℝ}}^{3}$ and $S^{3}$ denote the Euclidean space of dimension three and the sphere, respectively. $J_{\Theta }\left(\eta \right)$ is the transformation matrix. M, C(v), and D(v) represent the inertial, Coriolis-Centripetal and damping matrices, respectively. g(η) represents the restoring forces and moments.

A sliding mode trajectory tracking controller is designed to track the reference trajectory for USV as in [11]. All relevant position, orientation, linear and angular velocities, acceleration and forces describing the USV’s trajectory are calculated.

### 2.2. GPS Date Simulation

There are three kinds of measurements from GPS receiver, i.e., pesudorange, Doppler and carrier phase. In this subsection, we will introduce the measurement mode of pesudorange and Doppler. The first step is to develop the GPS constellation model, which can be used to generate the position of the satellites in the simulation. GPS archive data are available from the website of the International GNSS service (IGS). The next step is to model the signal transmission from the satellites. The main GPS measurement errors include ionospheric errors, tropospheric errors, etc. Further details can be found in [12]. Considering all of these aspects, the biased pseudorange measurement from one satellite vehicle at one instance is formulated as:

(4) $$\tilde{\rho }=\rho +c\left(t_{u}-t_{s}\right)+T_{iono}+T_{tropo}+\varepsilon_{\rho }$$

where $\tilde{\rho }$ denotes a measured range of user to satellite, ρ is a true range of user to satellite, $t_{u}$ and $t_{s}$ denote receiver clock bias and satellite clock bias, respectively, $T_{iono}$ and $T_{tropo}$ denote ionospheric delay and tropospheric delay, respectively, $\varepsilon_{\rho }$ denotes other un-modeled errors, i.e., multipath delay.

The relative motion between the satellite and the receiver results in change of the observed frequency of satellite signal. The Doppler can be used to estimate the user velocities from the satellite velocities. As in [3], the Doppler shift can be written as a projection of the relative velocity vector on the satellite line-of-sight vector:

(5) $$\tilde{\dot{\rho }}={\left(I_{u}^{s}\right)}^{T}\left(v_{s}-v_{u}\right)+c{\dot{t}}_{u}+\varepsilon_{\dot{\rho }}$$

where $I_{u}^{s}$ is the use-to-satellite line-of-sight unit vector, $v_{s}$ and $v_{u}$ represent the satellite and receiver velocity respectively. $I_{u}^{s}$ can be expressed as:

(6) $$I_{u}^{s}=\frac{1}{\left\|p_{s}-p_{u}\right\|}{\left[x_{s}-x_{u},y_{s}-y_{u},z_{s}-z_{u}\right]}^{T}$$

where $p_{s}=\left(x_{s},y_{s},z_{s}\right)$ and $p_{u}=\left(x_{u},y_{u},z_{u}\right)$ denote the position vector of satellite and receiver expressed in Earth-Central Earth Fixed (ECEF) coordinate.

### 2.3. IMU Date Simulation

For the purpose of simulating IMU data, raw measurements of accelerometer and gyroscope are needed. The trajectory simulator data are used as the basis in simulating the sensor data. Moreover, the velocity and angular rate data for the USVs are used.

The IMU measurements are provided by extracting the acceleration and angular velocity from the simulator model of USVs, which can be modeled as:

(7) $$f_{IMU}=R_{n}^{b}\left(\Theta \right)\left({\dot{v}}_{nb}^{n}-g_{n}\right)+f_{b}^{bias}+\varepsilon_{acc}^{b}$$

(8) $$\omega_{IMU}=\omega_{nb}^{b}+\omega_{b}^{bias}+\varepsilon_{gyro}^{b}$$

where $g_{n}$ represents gravity expressed in a navigation frame. $\varepsilon_{acc}^{b}$ and $\varepsilon_{gyro}^{b}$ denote the zero mean Gaussian distribute noise. $f_{b}^{bias}$ and $\omega_{b}^{bias}$ are bias errors of the specific forces and angular rate measurements, respectively. For the low cost MEMS-IMU, the sensor errors have the non-Gaussian characteristics. The bias errors in the gyroscope and accelerometer measurements in body frame will be transformed to be the position and velocity drifts in the navigation frame.

## 3. Quaternion-Based INS/GNSS Integration

### 3.1. Quaternion-Based Propagration and Observation Models

Define the vector form of quaternions as $q={\left[\begin{array}{cc} q_{0} & {\bar{q}}^{T} \end{array}\right]}^{T}$ with one real part $q_{0}$ and three imaginary parts given by the vector $\bar{q}={\left[\begin{array}{ccc} q_{1} & q_{2} & q_{3} \end{array}\right]}^{T}$. Quaternions are represented as a complex number with four bases and are used to compute the rotation from navigation frame to body frame. Based on Euler’s theorem, every change in the relative orientation of two rigid bodies or reference frame can be produced by means of a simple rotation from one frame to another along fixed axes. Given the invariant axis (rotation axis) and rotation angle (with magnitude ‖φ‖), the quaternion vector can be represented as:

(9) $$q=\left[\begin{array}{l} \cos\left(0.5\left\|\varphi \right\|\right)\\ \sin\left(0.5\left\|\varphi \right\|\right)u \end{array}\right],\;\text{with}\;u={\left[u_{x},u_{y},u_{z}\right]}^{T}$$

where u denotes the unit vector along the invariant axis. Apparently, q has the normality property, that is to say ‖q‖=1. It can be concluded that the quaternion vector has only three degrees of freedom, although q has four elements. Given a rotation vector $\varphi ={\left[\varphi_{1},\varphi_{2},\varphi_{3}\right]}^{T}$, the quaternion vector can be computed as:

(10) $$\left\|\varphi \right\|=\sqrt{\varphi_{1}^{2}+\varphi_{2}^{2}+\varphi_{3}^{2}},u=\frac{\varphi }{\left\|\varphi \right\|},q=\left[\begin{array}{l} \cos\left(0.5\left\|\varphi \right\|\right)\\ \sin\left(0.5\left\|\varphi \right\|\right)\frac{\varphi }{\left\|\varphi \right\|} \end{array}\right]$$

Conventionally, with a sufficiently small time interval, the quaternion vector is updated using vectors added together in discrete time domain. However, it should be noticed that the unit sphere defined by quaternion is not a Euclidean vector space. That is to say, the common definition of addition and scaling cannot be applied directly. In the AUPF algorithm, we will apply the quaternion product rule to update the quaternion vector. Thus, the system propagation model in discrete time domain can be expressed as:

(11) $$\begin{array}{l} p_{n,k+1}=p_{n,k}+v_{n,k}\cdot T\\ v_{n,k+1}=v_{n,k}+\left[R_{b}^{n}\left(q_{k}\right)f_{ib,k}^{b}+g_{n}\right]\cdot T\\ q_{k+1}=q_{k}⊗\left(\Delta q_{k}\right)\\ f_{b,k+1}^{bias}=f_{b,k}^{bias}+w_{f}\\ \omega_{b,k+1}^{bias}=\omega_{b,k}^{bias}+w_{\omega }\\ c\Delta t_{k+1}=c\Delta t_{k}+c\Delta {\dot{t}}_{k}\cdot T+w_{cbk}\\ c\Delta {\dot{t}}_{k+1}=c\Delta {\dot{t}}_{k}+w_{cdk} \end{array}$$

where ⊗ denotes the quaternion product. $p_{n,k}$ and $v_{n,k}$ denote the position and velocity in the navigation frame at epoch k. $g_{n}$ represents gravity expressed in navigation fame, which is assumed to be constant for local navigation. $c\Delta {\dot{t}}_{k}$ denotes the receiver clock drift error, which is modeled as a random walk process. $c\Delta t_{k}$ represents the range equivalent of the receiver clock bias, which is the integration of clock drift error. T is the system propagation time interval. $w_{f}$, $w_{\omega }$, $w_{cbk}$ and $w_{cdk}$ are Gaussian nose terms. $R_{b}^{n}\left(q\right)$ denotes the rotational transformation matrix from the body frame to the navigation frame, which can be formulated using the parameters of quaternion as:

(12) $$R_{b}^{n}=\left[\begin{array}{ccc} 1-2\left(q_{2}^{2}+q_{3}^{2}\right) & 2\left(q_{1}q_{2}-q_{0}q_{3}\right) & 2\left(q_{1}q_{3}+q_{0}q_{2}\right)\\ 2\left(q_{1}q_{2}+q_{0}q_{3}\right) & 1-2\left(q_{1}^{2}+q_{3}^{2}\right) & 2\left(q_{2}q_{3}-q_{0}q_{1}\right)\\ 2\left(q_{1}q_{3}-q_{0}q_{2}\right) & 2\left(q_{2}q_{3}+q_{0}q_{1}\right) & 1-2\left(q_{1}^{2}+q_{2}^{2}\right) \end{array}\right]$$

$\Delta q_{k}$ represents the quaternion rotation of body frame during the time interval:

(13) $$\Delta q_{k}=\left[\begin{array}{c} \cos\left(0.5\left\|\theta_{k}\right\|\right)\\ \sin\left(0.5\left\|\theta_{k}\right\|\right)\frac{\theta_{k}}{\left\|\theta_{k}\right\|} \end{array}\right]$$

where $\theta_{k}$ denotes the integral of the body frame angular rate measurements.

Equation (11) forms the propagation model of the system. In the tightly-coupled integration, the system observation model is formulated as Equation (14). Where j denotes the number of satellites in view, $I_{j}$ represents the estimated line-of-sight unit vector pointing from the initial estimate of the user position to the *j*-th satellite:

(14) $$\left[\begin{array}{c} {\hat{\rho }}_{1,k}-{\tilde{\rho }}_{1,k}\\ \vdots \\ {\hat{\rho }}_{j,k}-{\tilde{\rho }}_{j,k}\\ {\hat{\dot{\rho }}}_{1,k}-{\tilde{\dot{\rho }}}_{1,k}\\ \vdots \\ {\hat{\dot{\rho }}}_{j,k}-{\tilde{\dot{\rho }}}_{j,k} \end{array}\right]={\left[\begin{array}{ccccc} {\left(-I_{1,k}^{T}\right)}_{1\times 3} & 0_{1\times 3} & 0_{1\times 9} & 1 & 0\\ \vdots & \vdots & \vdots & \vdots & \vdots \\ {\left(-I_{j,k}^{T}\right)}_{1\times 3} & 0_{1\times 3} & 0_{1\times 9} & 1 & 0\\ 0_{1\times 3} & {\left(-I_{1,k}^{T}\right)}_{1\times 3} & 0_{1\times 9} & 0 & 1\\ \vdots & \vdots & \vdots & \vdots & \vdots \\ 0_{1\times 3} & {\left(-I_{j,k}^{T}\right)}_{1\times 3} & 0_{1\times 9} & 0 & 1 \end{array}\right]}_{2j\times 17}\cdot \left[\begin{array}{c} \delta p_{k}^{n}\\ \delta v_{k}^{n}\\ \delta \Psi_{k}\\ \delta f_{ib,k}^{b,error}\\ \delta \omega_{ib,k}^{b,error}\\ c\delta t_{k}\\ c\delta {\dot{t}}_{k} \end{array}\right]+\varepsilon_{k}$$

where ${\hat{\rho }}_{j,k}$ is the predicted pseudorange measurement from the *j*-th satellite, and:

(15) $${\hat{\rho }}_{j,k}=\sqrt{{\left(x_{j,k}^{n}-{\hat{x}}_{u,k}^{n}\right)}^{2}+{\left(y_{j,k}^{n}-{\hat{y}}_{u,k}^{n}\right)}^{2}+{\left(z_{j,k}^{n}-{\hat{z}}_{u,k}^{n}\right)}^{2}}+c\Delta {\hat{t}}_{k}$$

where ${\hat{x}}_{u,k}^{n}$, ${\hat{y}}_{u,k}^{n}$, ${\hat{z}}_{u,k}^{n}$ are the USV position estimates expressed in NED frame. $x_{j,k}^{n}$, $y_{j,k}^{n}$, $z_{j,k}^{n}$ are the *j*-th satellite position coordinates expressed in NED frame.

The predicted pseudorange-rate measurements are calculated as:

(16) $${\hat{\dot{\rho }}}_{j,k}=I_{j,k}^{n,n}\left(x_{j,k}^{n}-{\hat{x}}_{u,k}^{n}\right)+I_{j,k}^{n,e}\left(y_{j,k}^{n}-{\hat{y}}_{u,k}^{n}\right)+I_{j,k}^{n,d}\left(z_{j,k}^{n}-{\hat{z}}_{u,k}^{n}\right)+c\Delta {\hat{\dot{t}}}_{k}$$

where

$$\begin{array}{l} I_{j,k}^{n,n}=\left(x_{j,k}^{n}-{\hat{x}}_{u,k}^{n}\right)/{\hat{d}}_{j,k},I_{j,k}^{n,e}=\left(y_{j,k}^{n}-{\hat{y}}_{u,k}^{n}\right)/{\hat{d}}_{j,k},I_{j,k}^{n,d}=\left(z_{j,k}^{n}-{\hat{z}}_{u,k}^{n}\right)/{\hat{d}}_{j,k}\\ {\hat{d}}_{j,k}=\sqrt{{\left(x_{j,k}^{n}-{\hat{x}}_{u,k}^{n}\right)}^{2}+{\left(y_{j,k}^{n}-{\hat{y}}_{u,k}^{n}\right)}^{2}+{\left(z_{j,k}^{n}-{\hat{z}}_{u,k}^{n}\right)}^{2}} \end{array}$$

where ${\hat{\dot{\rho }}}_{j,k}$ is the predicted delta range measurement from the *j*-th satellite. ${\hat{\dot{x}}}_{u,k}^{n}$, ${\hat{\dot{y}}}_{u,k}^{n}$, ${\hat{\dot{z}}}_{u,k}^{n}$ are the USV velocity estimates expressed in NED frame. ${\dot{x}}_{j,k}^{n}$, ${\dot{y}}_{j,k}^{n}$, ${\dot{z}}_{j,k}^{n}$ are the *j*-th satellite velocity coordinates expressed in NED frame.

Due to introducing the quaternion, there exists dimension mismatch among the state vector and the state error covariance matrix. The reason is that the degree of freedom of a quaternion vector is three rather than four. When the state vector includes quaternion vector elements, the dimension of the state vector is 18 × 1; however, the dimension of the state error covariance matrix is 17 × 17. In order to solve the problem of dimension mismatch, we introduce the rotation vector in the rotation space, which is transformed from the corresponding quaternion vector error.

### 3.2. Quaternion-Based Integration Using UPF

The idea of UKF comes from the fact that it is much easier to approximate a Gaussian distribution, rather than to simulate a nonlinear function. The particles for UPF are generated using the UKF a posterior estimates. The state vector and its associated errors are defined as:

(17) $${\hat{x}}_{k}^{+}={\left[\begin{array}{c} {\hat{p}}_{n,k}^{+}\\ {\hat{v}}_{n,k}^{+}\\ {\hat{q}}_{k}^{+}\\ {\hat{f}}_{b,k}^{bias+}\\ {\hat{\omega }}_{b,k}^{bias+}\\ c\Delta {\hat{t}}_{k}^{+}\\ c\Delta {\hat{\dot{t}}}_{k}^{+} \end{array}\right]}_{18\times 1},\delta {\hat{x}}_{k}^{+}=\left[\begin{array}{c} {\hat{p}}_{n,k}^{+}-p_{n,k}\\ {\hat{v}}_{n,k}^{+}-v_{n,k}\\ {\hat{\varphi }}_{k}^{+}\\ {\hat{f}}_{b,k}^{bias+}-f_{b,k}^{bias}\\ {\hat{\omega }}_{b,k}^{bias+}-\omega_{b,k}^{bias}\\ c\Delta {\hat{t}}_{k}^{+}-c\Delta t_{k}\\ c\Delta {\hat{\dot{t}}}_{k}^{+}-c\Delta {\dot{t}}_{k} \end{array}\right]={\left[\begin{array}{c} \delta {\hat{p}}_{n,k}^{+}\\ \delta {\hat{v}}_{n,k}^{+}\\ {\hat{\varphi }}_{k}^{+}\\ \delta {\hat{f}}_{b,k}^{bias+}\\ \delta {\hat{\omega }}_{b,k}^{bias+}\\ c\delta {\hat{t}}_{k}^{+}\\ c\delta {\hat{\dot{t}}}_{k}^{+} \end{array}\right]}_{17\times 1}$$

where ${\hat{\varphi }}_{k}^{+}$ is the rotation vector corresponding to $q_{k}^{+}⊗{\left(q_{k}\right)}^{-1}$. The state error covariance $P_{k}^{+}$ can be formulated as:

(18) $$P_{k}^{+}={\left[\begin{array}{ccccccc} \sigma_{\delta \hat{p}}^{2} & 0_{3\times 3} & 0_{3\times 3} & 0_{3\times 3} & 0_{3\times 3} & 0_{3\times 3} & 0_{3\times 3}\\ 0_{3\times 3} & \sigma_{\delta \hat{v}}^{2} & 0_{3\times 3} & 0_{3\times 3} & 0_{3\times 3} & 0_{3\times 3} & 0_{3\times 3}\\ 0_{3\times 3} & 0_{3\times 3} & \sigma_{\delta \hat{\varphi }}^{2} & 0_{3\times 3} & 0_{3\times 3} & 0_{3\times 3} & 0_{3\times 3}\\ 0_{3\times 3} & 0_{3\times 3} & 0_{3\times 3} & \sigma_{\delta \hat{f}}^{2} & 0_{3\times 3} & 0_{3\times 3} & 0_{3\times 3}\\ 0_{3\times 3} & 0_{3\times 3} & 0_{3\times 3} & 0_{3\times 3} & \sigma_{\delta \hat{\omega }}^{2} & 0_{3\times 3} & 0_{3\times 3}\\ 0_{1\times 3} & 0_{1\times 3} & 0_{1\times 3} & 0_{1\times 3} & 0_{1\times 3} & \sigma_{c\delta \hat{t}}^{2} & 0\\ 0_{1\times 3} & 0_{1\times 3} & 0_{1\times 3} & 0_{1\times 3} & 0_{1\times 3} & 0 & \sigma_{c\delta \hat{\dot{t}}}^{2} \end{array}\right]}_{17\times 17}$$

The sigma-points are generated according to Equation (19)

(19) $$\begin{array}{l} {\hat{x}}_{k-1,i}^{+}={\hat{x}}_{k-1}^{+}+{\left(\sqrt{nP_{k-1}^{+}}\right)}_{i}^{T},i=1,\ldots ,n\\ {\hat{x}}_{k-1,i+n}^{+}={\hat{x}}_{k-1}^{+}-{\left(\sqrt{nP_{k-1}^{+}}\right)}_{i}^{T},i=1,\ldots ,n \end{array}$$

where 2n(n=17) equally weighted sigma-points are generated. The calculated sigma-points has the form of:

(20) $${\hat{x}}_{k-1,i}^{+}=\left[\begin{array}{c} {\hat{p}}_{n,k-1}^{+}+\Delta {\hat{p}}_{n,k-1,i}^{+}\\ {\hat{v}}_{n,k-1}^{+}+\Delta {\hat{v}}_{n,k-1,i}^{+}\\ \delta q\left({\hat{\varphi }}_{k-1,i}^{+}\right)⊗{\hat{q}}_{k-1}^{+}\\ {\hat{f}}_{b,k-1}^{bias+}+\Delta {\hat{f}}_{b,k-1,i}^{bias+}\\ {\hat{\omega }}_{b,k-1}^{bias+}+\Delta {\hat{\omega }}_{b,k-1,i}^{bias+}\\ c\Delta {\hat{t}}_{k-1}^{+}+\Delta c\Delta {\hat{t}}_{k-1,i}^{+}\\ c\Delta {\hat{\dot{t}}}_{k-1}^{+}+\Delta c\Delta {\hat{\dot{t}}}_{k-1,i}^{+} \end{array}\right],{\hat{x}}_{k-1,i+n}^{+}=\left[\begin{array}{c} {\hat{p}}_{n,k-1}^{+}-\Delta {\hat{p}}_{n,k-1,i}^{+}\\ {\hat{v}}_{n,k-1}^{+}-\Delta {\hat{v}}_{n,k-1,i}^{+}\\ \delta q\left(-{\hat{\varphi }}_{k-1,i}^{+}\right)⊗{\hat{q}}_{k-1}^{+}\\ {\hat{f}}_{b,k-1}^{bias+}-\Delta {\hat{f}}_{b,k-1,i}^{bias+}\\ {\hat{\omega }}_{b,k-1}^{bias+}-\Delta {\hat{\omega }}_{b,k-1,i}^{bias+}\\ c\Delta {\hat{t}}_{k-1}^{+}-\Delta c\Delta {\hat{t}}_{k-1,i}^{+}\\ c\Delta {\hat{\dot{t}}}_{k-1}^{+}-\Delta c\Delta {\hat{\dot{t}}}_{k-1,i}^{+} \end{array}\right]$$

Define the delta rotation angle rate in the quaternion form as:

(21) $$\delta q\left({\hat{\varphi }}_{k-1,i}^{+}\right)=\left[\begin{array}{c} \cos\left(0.5\left\|{\hat{\varphi }}_{k-1,i}^{+}\right\|\right)\\ \sin\left(0.5\left\|{\hat{\varphi }}_{k-1,i}^{+}\right\|\right)\frac{{\hat{\varphi }}_{k-1,i}^{+}}{\left\|{\hat{\varphi }}_{k-1,i}^{+}\right\|} \end{array}\right]$$

The a priori mean and the state error covariance matrix are computed through Equations (22) and (23):

(22) $${\hat{x}}_{k}^{-}=\frac{1}{2n}\sum_{i=1}^{2n}f_{k-1}\left({\hat{x}}_{k-1,i}^{+}\right)$$

(23) $$P_{k}^{-}=\frac{1}{2n}{\sum_{i=1}^{2n}\left[f_{k-1}\left({\hat{x}}_{k-1,i}^{+}\right)-{\hat{x}}_{k}^{-}\right]\left[f_{k-1}\left({\hat{x}}_{k-1,i}^{+}\right)-{\hat{x}}_{k}^{-}\right]}^{T}+Q_{k-1}$$

The same as in Equation (19), we generate the sigma-points for measurements update by replacing $P_{k-1}^{+}$ with $P_{k}^{-}$. The sigma-points ${\hat{x}}_{k,i}^{-}$ are calculated according to Equation (20). With the sigma-points propagation, the predicted measurement and covariance matrix are calculated according to Equations (24)–(26):

(24) $${\hat{y}}_{k}=\frac{1}{2n}\sum_{i=1}^{2n}h_{k}\left({\hat{x}}_{k,i}^{-}\right)$$

(25) $$P_{k}^{yy}=\frac{1}{2n}{\sum_{i=1}^{2n}\left[h_{k}\left({\hat{x}}_{k,i}^{-}\right)-{\hat{y}}_{k}\right]\left[h_{k}\left({\hat{x}}_{k,i}^{-}\right)-{\hat{y}}_{k}\right]}^{T}+R_{k}$$

(26) $$P_{k}^{xy}=\frac{1}{2n}{\sum_{i=1}^{2n}\left[{\hat{x}}_{k,i}^{-}-{\hat{x}}_{k}^{-}\right]\left[h_{k}\left({\hat{x}}_{k,i}^{-}\right)-{\hat{y}}_{k}\right]}^{T}$$

At each measurement epoch of GPS, we update the state estimate and the covariance as KF frame:

(27) $$\begin{array}{l} K_{k}=P_{k}^{xy}{\left(P_{k}^{yy}\right)}^{-1}\\ {\hat{x}}_{k}^{+}={\hat{x}}_{k}^{-}+K_{k}\left({\tilde{y}}_{k}-{\hat{y}}_{k}\right)\\ P_{k}^{+}=P_{k}^{-}-K_{k}P_{k}^{yy}K_{k}^{T} \end{array}$$

For the UPF, the a posterior estimates of ${\hat{x}}_{k}^{+}$ and $P_{k}^{+}$ from Equation (27) are used to form the importance density distribution for generating particles:

(28) $$\chi_{k,i}^{+}={\hat{x}}_{k}^{+}+\Delta {\hat{x}}_{k,i}^{+}=\left[\begin{array}{c} {\hat{p}}_{n,k}^{+}+\Delta {\hat{p}}_{n,k,i}^{+}\\ {\hat{v}}_{n,k}^{+}+\Delta {\hat{v}}_{n,k,i}^{+}\\ \delta q\left({\hat{\varphi }}_{k,i}^{+}\right)⊗{\hat{q}}_{k}^{+}\\ {\hat{f}}_{b,k}^{bias+}+\Delta {\hat{f}}_{b,k,i}^{bias+}\\ {\hat{\omega }}_{b,k}^{bias+}+\Delta {\hat{\omega }}_{b,k,i}^{bias+}\\ c\Delta {\hat{t}}_{k}^{+}+\Delta c\Delta {\hat{t}}_{k,i}^{+}\\ c\Delta {\hat{\dot{t}}}_{k}^{+}+\Delta c\Delta {\hat{\dot{t}}}_{k,i}^{+} \end{array}\right],\Delta {\hat{x}}_{k,i}^{+}∼N\left(0,P_{k}^{+}\right)$$

where i=1,⋯,N, N is the number of particles. The normalized importance weights are computed as:

(29) $$w\left(\chi_{k,i}^{+}\right)=\frac{p\left({\tilde{y}}_{k}|\chi_{k,i}^{+}\right)N\left(\chi_{k,i}^{+};{\hat{x}}_{k}^{-},P_{k}^{-}\right)}{N\left(\chi_{k,i}^{+};{\hat{x}}_{k}^{+},P_{k}^{+}\right)}$$

(30) $$\bar{w}\left(\chi_{k,i}^{+}\right)=\frac{w\left(\chi_{k,i}^{+}\right)}{\sum_{i=1}^{N}w\left(\chi_{k,i}^{+}\right)},\text{with}\;\sum_{i=1}^{N}\bar{w}\left(\chi_{k,i}^{+}\right)=1$$

where

$$p\left({\tilde{y}}_{k}|\chi_{k,i}^{+}\right)=\frac{1}{\sqrt{{\left(2\pi \right)}^{m}\left\|R_{k}\right\|}}\text{exp}\left\{-\frac{{\left[{\tilde{y}}_{k}-h_{k}\left(\chi_{k,i}^{+}\right)\right]}^{T}R^{-1}\left[{\tilde{y}}_{k}-h_{k}\left(\chi_{k,i}^{+}\right)\right]}{2}\right\}$$

$$N\left(\chi_{k,i}^{+};{\hat{x}}_{k}^{-},P_{k}^{-}\right)=\frac{1}{\sqrt{{\left(2\pi \right)}^{n}\left\|P_{k}^{-}\right\|}}\text{exp}\left\{-\frac{{\left[\chi_{k,i}^{+}-{\hat{x}}_{k}^{-}\right]}^{T}{\left(P_{k}^{-}\right)}^{-1}\left[\chi_{k,i}^{+}-{\hat{x}}_{k}^{-}\right]}{2}\right\}$$

$$N\left(\chi_{k,i}^{+};{\hat{x}}_{k}^{+},P_{k}^{+}\right)=\frac{1}{\sqrt{{\left(2\pi \right)}^{n}\left\|P_{k}^{+}\right\|}}\text{exp}\left\{-\frac{{\left[\chi_{k,i}^{+}-{\hat{x}}_{k}^{+}\right]}^{T}{\left(P_{k}^{+}\right)}^{-1}\left[\chi_{k,i}^{+}-{\hat{x}}_{k}^{+}\right]}{2}\right\}$$

where m denotes the observer dimension which varies during the time and is based on the number of satellites being tracked by the receiver.

The a posteriori mean and covariance estimates are computed as:

(31) $$\begin{array}{l} {\hat{x}}_{k}^{+}=\sum_{i=1}^{p}\bar{w}\left(\chi_{k,i}^{+}\right)\chi_{k,i}^{+}\\ P_{k}^{+}=\sum_{i=1}^{N}\bar{w}\left(\chi_{k,i}^{+}\right)\left[\chi_{k,i}^{+}-{\hat{x}}_{k}^{+}\right]{\left[\chi_{k,i}^{+}-{\hat{x}}_{k}^{+}\right]}^{T} \end{array}$$

where $\chi_{k,i}^{+}-{\hat{x}}_{k}^{+}$ can be calculated as:

$${\left[{\left({\hat{p}}_{n,k,i}^{+}-{\hat{p}}_{n,k}^{+}\right)}^{T},{\left({\hat{v}}_{n,k,i}^{+}-{\hat{v}}_{n,k}^{+}\right)}^{T},{\left({\hat{\varphi }}_{k,i}^{+}\right)}^{T},{\left({\hat{f}}_{b,k,i}^{bias+}-{\hat{f}}_{b,k}^{bias+}\right)}^{T},{\left({\hat{\omega }}_{b,k,i}^{bias+}-{\hat{\omega }}_{b,k}^{bias+}\right)}^{T},{\left(c\Delta {\hat{t}}_{k,i}^{+}-c\Delta {\hat{t}}_{k}^{+}\right)}^{T},{\left(c\Delta {\hat{\dot{t}}}_{k,i}^{+}-c\Delta {\hat{\dot{t}}}_{k}^{+}\right)}^{T}\right]}^{T}.$$

${\hat{\varphi }}_{k,i}^{+}$ is the rotation vector corresponding to ${\hat{q}}_{k,i}^{+}⊗{\left({\hat{q}}_{k}^{+}\right)}^{-1}$. Due to the fact that the unit quaternion is not mathematically closed for addition and scalar multiplication, a normalization procedure is necessary to ensure the constraint $q^{T}q=1$ is satisfied in the presence of measurement noise and numerical round-off errors. The normalization can be conducted by replacing ${\hat{q}}_{k}^{+}$ with ${\hat{q}}_{k}^{+}/\left\|{\hat{q}}_{k}^{+}\right\|$.

### 3.3. Residual-Based AUPF

For the a posterior state estimations, a reliable resolution is proposed which greatly depends on prior statistics of the system process and measurement noise. However, these statistics are hardly known exactly in practice because they are based on the types of applications and process dynamics [13]. In addition, the estimation environment regarding INS/GNSS kinematic application is not always fixed but subject to change [14]. In order to maintain the estimation accuracy, a residual-based covariance matching technique is used to adaptively adjust the algorithm parameters and to track the changes in the noise source. For the INS/GNSS integration, the IMU measurements are used in system model, and the product manual specifies the sensor turn-on bias, temperature related variations in biases and noises. In this paper, we assume that the process noise error covariance Q is approximately known, and we focus on the adaptive estimation of sensor measurement error covariance $\hat{R}$.

Different from [2], which proposed an adaptive EKF algorithm, for the UKF, modified measurement noise error covariance matrices are adaptively updated as:

(32) $${\hat{R}}_{k}={\hat{C}}_{v_{k}}+P_{k}^{yy+},\;\text{where}\;{\hat{C}}_{v_{k}}=\frac{1}{N}\sum_{j=j_{0}}^{k}z_{j}z_{j}^{T}$$

where $P_{k}^{yy+}$ is calculated as:

(33) $$P_{k}^{yy+}=\frac{1}{2n}\sum_{i=1}^{2n}\left[h_{k}\left({\hat{x}}_{k,i}^{+}\right)-{\hat{y}}_{k}^{+}\right]{\left[h_{k}\left({\hat{x}}_{k,i}^{+}\right)-{\hat{y}}_{k}^{+}\right]}^{T}$$

where ${\hat{y}}_{k}^{+}=\frac{1}{2n}\sum_{i=1}^{2n}h_{k}\left({\hat{x}}_{k,i}^{+}\right)$. The ${\hat{x}}_{k,i}^{+}$ denotes the sigma-points drawn from the UKF a posterior estimates. The sigma-points are generated as:

(34) $$\begin{array}{l} {\hat{x}}_{k,i}^{+}={\hat{x}}_{k}^{+}+{\left(\sqrt{nP_{k}^{+}}\right)}_{i}^{T},i=1,\ldots ,n\\ {\hat{x}}_{k,i+n}^{+}={\hat{x}}_{k}^{+}-{\left(\sqrt{nP_{k}^{+}}\right)}_{i}^{T},i=1,\ldots ,n \end{array}$$

(35) $${\hat{x}}_{k,i}^{+}=\left[\begin{array}{c} {\hat{p}}_{n,k}^{+}+\Delta {\hat{p}}_{n,k,i}^{+}\\ {\hat{v}}_{n,k}^{+}+\Delta {\hat{v}}_{n,k,i}^{+}\\ \delta q\left({\hat{\varphi }}_{k,i}^{+}\right)⊗{\hat{q}}_{k}^{+}\\ {\hat{f}}_{b,k}^{bias+}+\Delta {\hat{f}}_{b,k,i}^{bias+}\\ {\hat{\omega }}_{b,k}^{bias+}+\Delta {\hat{\omega }}_{b,k,i}^{bias+}\\ c\Delta {\hat{t}}_{k}^{+}+\Delta c\Delta {\hat{t}}_{k,i}^{+}\\ c\Delta {\hat{\dot{t}}}_{k}^{+}+\Delta c\Delta {\hat{\dot{t}}}_{k,i}^{+} \end{array}\right],{\hat{x}}_{k,i+n}^{+}=\left[\begin{array}{c} {\hat{p}}_{n,k}^{+}-\Delta {\hat{p}}_{n,k,i}^{+}\\ {\hat{v}}_{n,k}^{+}-\Delta {\hat{v}}_{n,k,i}^{+}\\ \delta q\left(-{\hat{\varphi }}_{k,i}^{+}\right)⊗{\hat{q}}_{k}^{+}\\ {\hat{f}}_{b,k}^{bias+}-\Delta {\hat{f}}_{b,k,i}^{bias+}\\ {\hat{\omega }}_{b,k}^{bias+}-\Delta {\hat{\omega }}_{b,k,i}^{bias+}\\ c\Delta {\hat{t}}_{k}^{+}-\Delta c\Delta {\hat{t}}_{k,i}^{+}\\ c\Delta {\hat{\dot{t}}}_{k}^{+}-\Delta c\Delta {\hat{\dot{t}}}_{k,i}^{+} \end{array}\right]$$

The calculated sigma-points have the form of Equation (35). The delta rotation angle rate in the quaternion form can be formulated as:

(36) $$\delta q\left({\hat{\varphi }}_{k,i}^{+}\right)=\left[\begin{array}{c} \cos\left(0.5\left\|{\hat{\varphi }}_{k,i}^{+}\right\|\right)\\ \sin\left(0.5\left\|{\hat{\varphi }}_{k,i}^{+}\right\|\right)\frac{{\hat{\varphi }}_{k,i}^{+}}{\left\|{\hat{\varphi }}_{k,i}^{+}\right\|} \end{array}\right]$$

**Figure 2:**
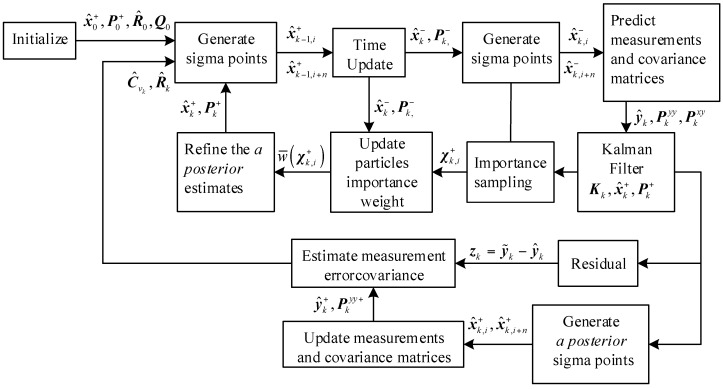
Adaptive unscented particle filter (AUPF) algorithm flowchart.

The flowchart of the proposed AUPF is illustrated in Figure 2.

## 4. Simulation Results

**Figure 3:**
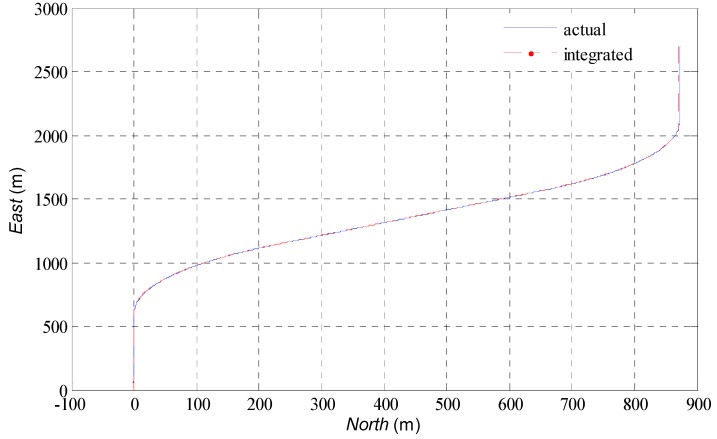
The trajectory of the USV.

A simulator with the module of USV trajectory simulation and the raw measurements simulation for IMU and GPS was developed. The trajectory used in this simulation scenario is illustrated in Figure 3. We assume that the USV is operating at sea level. The sliding mode controller in [15] drives the USV fallowing the designed trajectory. To represent a real MEMS-IMU as closely as possible, different error types including the Gaussian model and random bias were induced in the simulated IMU. Raw GPS pseudorange and Doppler measurements were simulated at a 1 Hz rate, while the IMU raw data were simulated at a 100 Hz rate. The sensor error characteristics of MEMS-IMU are given in Table 1. The program is implemented in MATLAB R2013a using Intel Xeon E31270 CPU and 8 GB RAM.

**Figure 4:**
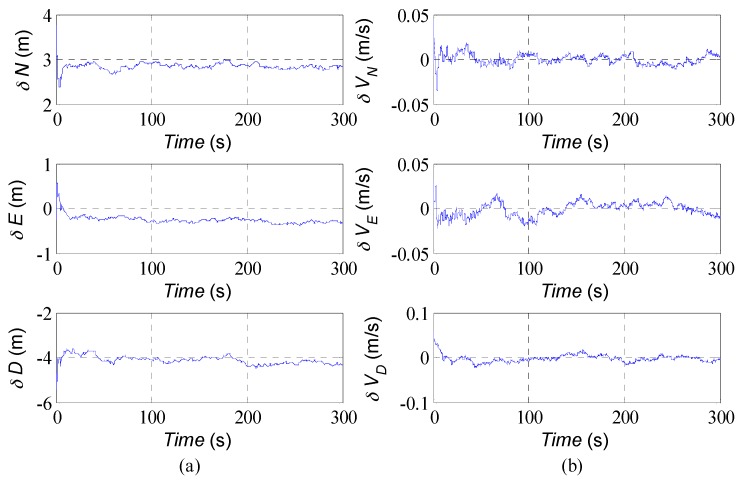
(**a**) estimation errors of the position; (**b**) estimation errors of the velocity.

**Figure 5:**
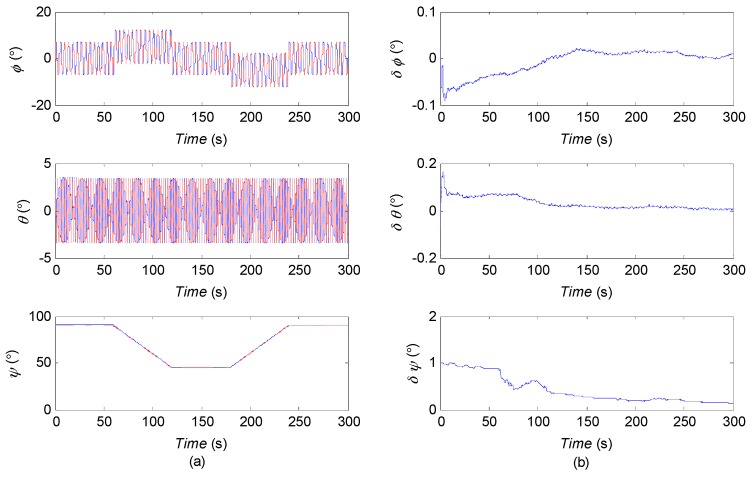
(**a**) roll, pitch and yaw of the USV; (**b**) estimation of the attitude errors (transformed from quaternion to corresponding Euler angles).

**Figure 6:**
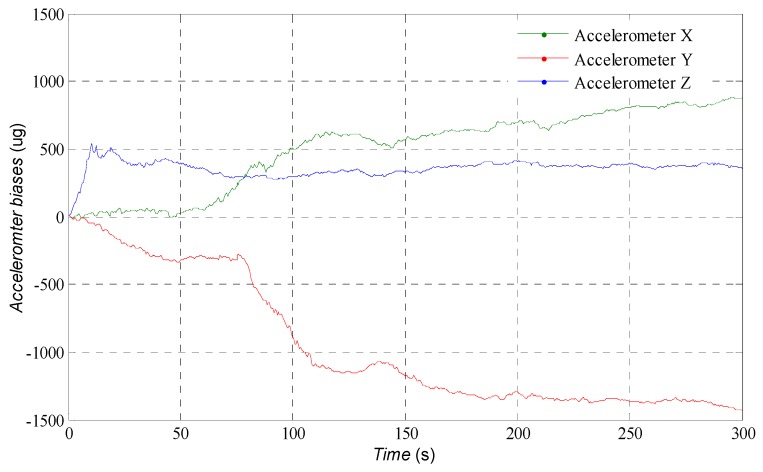
Estimation of the accelerometer bias.

**Figure 7:**
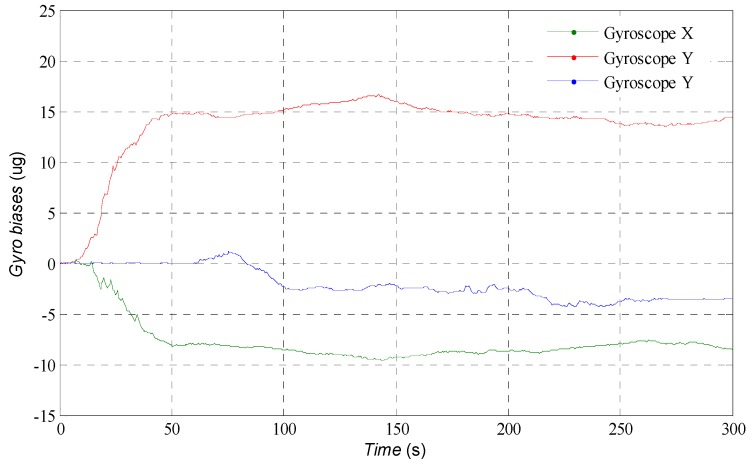
Estimation of the gyroscope bias.

Figure 3 shows the estimation results of trajectory of USV in horizontal plane. Although the low cost MEMS-IMU is used, the agreement with the actual trajectory is generally good. The estimated position and velocity errors can be found in Figure 4. After the initial transient, the estimation error in the north direction remains less than 3 m approximately, 0.5 m in the east direction and −4.2 m in the down direction; the errors of velocity remain within a region of approximately ±0.05 m/s. In Figure 5, the attitudes estimated by the AUPF algorithm (using quaternion) are plotted. The quaternion are converted to their corresponding Euler angles. Except the initial transient, the roll and pitch errors remain mostly in the region ±0.1 deg, but the yaw error tends to be a little larger. Accelerometer bias estimation results are illustrated in Figure 6. Gyroscope bias estimation results are plotted in Figure 7. Due to the fact that the yaw angle is the least observable state in practical vehicle motion, we can see form Figure 7 that the bias error on the *z*-axis takes more time to be stable.

**Figure 8:**
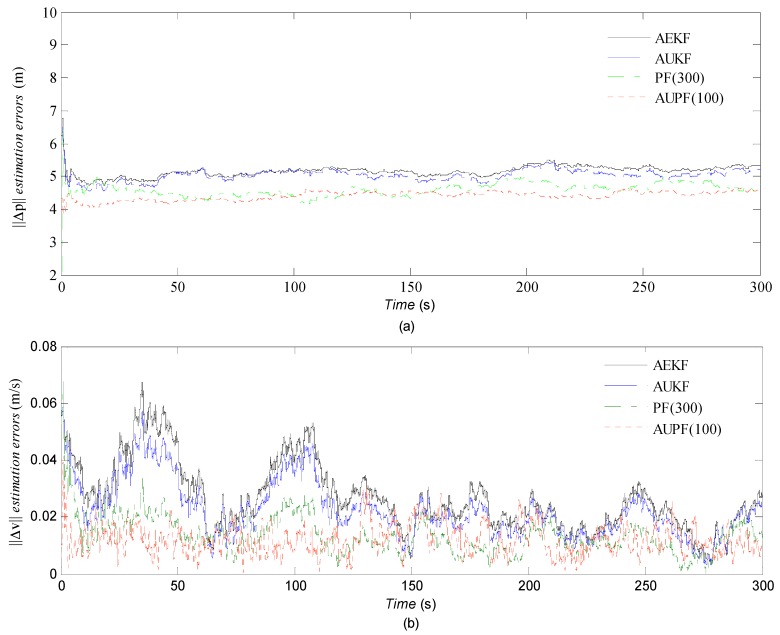
(**a**) Norm of position estimation errors; (**b**) Norm of velocity estimation errors.

In order to illustrate the performance of the proposed AUPF algorithm, several groups of tests adopting the AEKF, AUKF, PF and AUPF are conducted, respectively, in this part. For comparing the navigation accuracy among the algorithms mentioned above, we choose the norm of the position (‖Δp‖) and velocity (‖Δv‖) estimation errors for analysis, which are calculated as $\left\|\Delta x\right\|=\sqrt{\Delta x_{N}^{2}+\Delta x_{E}^{2}+\Delta x_{D}^{2}}$. As shown in Figure 8, AUPF presents best estimation accuracy compared with others.

Furthermore, with the same group of GPS and IMU data, the estimation accuracy and computation time of the algorithms are illustrated in Table 2. M(‖Δp‖) and M(‖Δv‖) denote the mean of the norm of the position and velocity estimation errors. V(‖Δp‖) and V(‖Δv‖) denote the variance of the norm of the position and velocity estimation errors. The time represents the algorithm processing time, which depends on the computation power of the computer and only for reference. We conduct one simulation run for AEKF and AUKF because the same prior statistical parameters and the same group of sensor data result in unchanged performance. For the PF based algorithm, the particles are generated randomly, so the estimation results have slight differences after each simulation. Table 2 shows the average values of 100 runs of the algorithms. We can see that AUPF (100 particles) can significantly improve the accuracy compared with PF (300 particles), and with computational efficiency at the same time.

Although the validation of the proposed AUPF algorithm is conducted from simulation in this paper, it has a theoretical foundation with a stringent mathematical representation. However, a real data experiment is an intuitively clear way to validate the performance of the presented AUPF algorithm, and it is of great practical interest to validate and compare it with other filters in experiments.

## 5. Conclusions

In this paper, we have studied the problem of navigation system design for low-cost USVs, in which we mainly focus on the filtering algorithm. A quaternion-based tightly-coupled integration approach is chosen to fuse the low-cost MEMS-INS measurements and GPS data. Due to the nonlinear and non-Gaussian properties of the INS/GNSS tightly-coupled integration problem, an APUF algorithm is proposed, which combines UKF with PF. A residual-base covariance matching technique is used to adaptively adjust the parameters and to track the changes in observation data in an adaptive manner. The resulting AUPF algorithm can reduce the computational burden without degrading the system estimation accuracy. A USV trajectory simulator is designed to generate the raw senor measurements of IMU and GNSS. The simulation results verify the robustness and accuracy of the AUPF algorithm in comparison to the AEKF and AUKF. In addition, the comparison with PF underlines the computational advantage of AUPF.

## Figures and Tables

**Table 1:** Error characteristics of the simulated MEMS-IMU.

Gyroscope (Angular Rates)		Accelerometer (Specific Forces)	
Bias in-run Stability	≦±13 [°/h] (1σ)	Bias in-run Stability	≦±1300 [μg] (1σ)
Noise (ARW)	0.028 [°/s/√Hz] (1σ)	Noise (VRW)	70 [μg/√Hz] (1σ)
Scale Factor Error	<1000 [ppm]	Scale Factor Error	<1000 [ppm]

Micro-electromechanical system inertial measurement unit (MEMS-IMU).

**Table 2:** Performance comparison between state-of-the-art algorithms.

Algorithm	Runs	M(‖Δp‖) [m]	V(‖Δp‖) [m]	M(‖Δv‖) [m/s]	V(‖Δv‖) [m/s]	Time [s]
AEKF	1	5.2451	0.0426	0.0327	0.0042	3.3774
AUKF	1	5.1962	0.0397	0.0286	0.0037	17.3859
PF(300)	100	4.5173	0.0315	0.0207	0.0021	283.4482
AUPF(100)	100	4.2358	0.0273	0.0183	0.0014	62.1538

Adaptive extended Kalman filter (AEKF); Adaptive unscented Kalman filter (AUKF); Particle filter (PF); Adaptive unscented particle filter (AUPF).
